# FITLIGHT Training and Its Influence on Visual-Motor Reactions and Dribbling Speed in Female Basketball Players: Prospective Evaluation Study

**DOI:** 10.2196/70519

**Published:** 2025-07-04

**Authors:** Ahmed K Hassan

**Affiliations:** 1 Department of Physical Education, College of Education, King Faisal University, P.O. Box 400, Al-Ahsa, 31982, Saudi Arabia, 966 536980427; 2 Department of Team Sports and Racket Games, Faculty of Physical Education, Minia University, Minia, Egypt

**Keywords:** reaction time, reactive time, FITLIGHT technology, visual-motor interaction, sports performance

## Abstract

**Background:**

Basketball exists as a team-based sport played on a court involving intense physical demands because players need continuous movement between offensive and defensive zones.

**Objective:**

The aim of the current research was to investigate the impact of a training program implementing FITLIGHT (FITLIGHT Corp) technology on female basketball players’ visual-motor interactions and dribbling speed.

**Methods:**

The study included 28 female basketball players. Participants were divided into 2 groups, experimental group (EG; n=14, mean age 18.29, SD 0.99 y; mean height 174.50, SD 2.10 cm; mean weight 75.36, SD 2.10 kg; mean training 5.64, SD 0.50 y) and control group (CG; n=14, mean age 18.50, SD 0.65 y; mean height 175.64, SD 1.55 cm; mean weight 75.57, SD 1.79 kg; mean training 5.43, SD 0.51 y), in a random manner. Pre- and post-measurements were used, and FITLIGHT training was conducted for 10 weeks with 4 sessions per week. The FITLIGHT training program targeted the elements of visual-motor interactions and dribbling speed for the EG. The CG experienced the same training regimen, but did not participate in the FITLIGHT training. The tests that were adopted and administered were the reaction time in the right hand and left hand, the reactive time (*t* test), and the reactive time with dribbling (*t* test D) tests. Statistical analysis included the calculation of descriptive statistics for minimum and maximum values and mean and SD, which were used for paired *t* tests for within-group comparison alongside independent *t* tests for between-group differences. Effect sizes (Cohen *d*) measured small effects at less than 0.2, medium effects at 0.2‐0.5, and large effects at greater than 0.8 at *P*<.05.

**Results:**

All the EG improvements were statistically significant (*P*<.001), with reaction time in the right hand improving by 0.04 ms more than that in CG (0.016 ms; effect sizes *d*=0.87 for the EG and *d*=0.79 for the CG). The reaction time in the left hand of the EG was 0.05 ms faster (*d*=0.97), compared with a difference of 0.019 ms in the CG (*d*=0.71). Participants in the EG had 1.90 seconds more reactive time (*d*=0.98) than those in the CG, who had 0.85 seconds (*d*=0.97). For dribbling in the EG, reactive time improved by 4.08 seconds (*d*=0.98), much better than the improvement seen in the CG of 1.71 seconds (*d*=0.93) when compared with using effect sizes. The analysis of the data is finished, showing that the EG had much larger effects than the CG. Study participants achieved large effect sizes during reaction time in the right hand (*d*=0.51), reaction time in the left hand (*d*=0.71), reactive time (*d*=0.84), and reactive time with dribbling (*d*=0.88).

**Conclusions:**

A pairwise comparison between the EG and the CG also revealed a statistically significant difference in the EG, which proved that the EG outperformed the CG. This study provides evidence for the enhanced visual-motor interactions and dribbling speeds of female basketball players who received training that included the use of the FITLIGHT technique.

## Introduction

Basketball is a team sport that is played on a court. The activity is extremely strenuous, particularly due to the constant movement between offense and defense, and entails highly energetic movements such as jumping, running, and shuffling [1]. Considering the grueling nature of the exercises involved in basketball, it is appropriate for coaches to provide the essential measures to monitor the physical pressures that players experience [2]. Montella et al [3] confirmed that, as a team sport, basketball is quantitative in nature due to the elements of performance, strength, and endurance, as the focus is on the statistical and operational dimensions of quality. Basketball may be characterized as a somewhat intermittent team sport in which different players’ movements and activities are performed repeatedly in the form of short bouts of high intensity followed by periods of low to moderate intensity [1,4]. Modern basketball can be described as a game that involves both rhythm and skills [5], as shown in the global level of honed skills observed across different basketball tournaments, revisiting current trends in training young talents [6].

FITLIGHT (FITLIGHT Corp) training is a modern trend in the training of sportspeople. It involves the use of wireless light units connected to a tablet to promote thinking processes and motor skills. This technology has been incorporated into several sports due to the improvements in the athletes and in their mental functions. After conducting an analysis of judo athletes, the researcher demonstrated that a 5-week cognitive-motor training program using FITLIGHT enhanced their executive functions, their cognition, and their motor skills [7]. The FITLIGHT training system also demonstrated acceptable intertest reactivity for the assessment of reaction times. This is a crucial parameter for the evaluation of cognitive states following traumatic brain injury [8]. A study of basketball players showed that small side games using FITLIGHT training enhanced abilities, basic skills, and performances in the experimental group (EG) [9]. FITLIGHT technology helped to provide feedback during training, thus improving the athletes’ strategies and their ability to correct their own techniques in the same way that a personal trainer would do [10]. FITLIGHT is a training tool that can be used to improve basketball players’ physical skills and visual development [11]. FITLIGHT includes an internal contact sensor that can be used to turn each light unit off or on. These lights can be switched on individually or several at a time, with the option of altering the patterns and durations of the stimulation (FITLIGHT Corp). In addition to their design that enables different light sequences, it is possible to mount the FITLIGHT to walls, columns, floors, and other training equipment [12].

The nature of performance in basketball requires the integration of motor and hand-eye coordination (visual training) with the aim of enhancing interactions at the level of visualization skills, developing skills, and increasing the level of focused attention. This link is authentic and mimics the dynamics of an actual player during a game. The faster a player becomes aware of the ball, their teammates, or the space on the field, the better and faster they are at making decisions and executing them [13]. Visualization via modern techniques and instruments has a positive effect on the enhancement of visual neural networks and some cerebral functions. Visual training programs also improve athletic performances and decrease weaknesses by sharpening hand-eye coordination, decreasing reaction times, expanding peripheral vision, and enhancing the playing field [14]. The researcher noted that basketball coaches hardly considered the aspect of hand speed, which made it easier for the opposing team to counter and score points or to score in the basket because basketball requires extensive hand-eye coordination with regard to receiving, passing, dribbling, and shooting the ball at the basket. It also requires rapid visual reactions in switching between the attacking and the defending lines, as previous studies have indicated [1,9,11-22]. Thus, the researcher conducted a survey study of the tests and then confirmed the poor performances of players in those tests; hence, there was the need to undertake a study to determine the impact of FITLIGHT exercises on enhancing the visual-motor coordination and dribbling speeds of female basketball players to assist coaches in understanding the correct approaches and strategies for enhancing their performance levels.

This study aimed to look at female basketball players’ visual-motor coordination after a 10-week FITLIGHT-based training program, by using the Lafayette Instrument 63035A Response Panel, and in addition, to evaluate in skillful dribbling speed with the *t* test protocol. The methodology focused on a 10-week intervention with 4 weekly sessions, designed to optimize skill acquisition through structured, repetitive practice and real-time feedback mechanisms [10]. This approach diverges from previous studies that used shorter, less frequent training protocols. To quantify outcomes, visual-motor coordination was assessed using standardized reaction time and hand-eye coordination tests, while dribbling speed improvements were measured via timed obstacle courses and motion analysis software [7,9,11,21,22]. These metrics align with kinematic analysis, a validated tool for evaluating movement patterns and comparing performance across interventions [6]. The study’s design builds on recent advancements in digital training technologies. By extending the training duration to 10 weeks, this research creates conditions to assess long-term skill development, contrasting with previous work that often focused on short-term interventions. In addition, the exclusive focus on female athletes addresses a critical gap in sports science literature, where gender-specific adaptations to training protocols remain underexplored.

We hypothesized that females in the EG would show significantly improved visual-motor coordination and dribbling speed compared with those in the control group (CG), as measured at the beginning and end of the intervention. In addition, we hypothesized that the prolonged practice period would make FITLIGHT more efficient, so the differences in dribbling speed between the EG and CG after measurement would favor the EG, because of better visual-motor coordination. This structure highlights how research in this area helps improve the training of women athletes, provides practical guidance for coaches and practitioners, and advances the science surrounding skill retention and performance when using technologies.

## Methods

### Study Design

The research design implemented a FITLIGHT system training protocol, which ran for 10 weeks to enhance visual-motor coordination ability and dribbling speed for Al-Way Club’s female basketball team players from Minya Governorate. Furthermore, 2 research groups formed the EG and CG. The EG participated in FITLIGHT-based training, whereas the CG executed the traditional program. G*Power software from the University of Düsseldorf enabled statistical power analysis to determine the needed sample size [23], which became 28 amateur participants after excluding 6 people from the initial group of 34 participants because of certain exclusion criteria. The researchers strictly chose female participants who had more than 4 years of sports experience and commitment to assessment and training activities. The first round of evaluation, known as pretests, was administered to participants on August 4 and August 5, 2024. The research relied on dependable assessment instruments, including the visual response board and the FITLIGHT technology kit for data collection. The training program lasted from August 6 to October 13, 2024. The experimental participants underwent FITLIGHT training throughout 10 weeks in 4 scheduled sessions, yet the CG underwent conventional training without access to FITLIGHT devices. The supervisors monitored the gradual and timed progress of both the intensity level and duration while ensuring safety, along with correct performance by participants throughout the protocol. Both posttests took place from October 15 to October 16, 2024, according to the same assessment process as the initial tests. The study participants achieved satisfactory statistical significance due to their 28-person sample, which yielded an effect power of 0.81 through a power analysis.

### Participants

An experimental approach was employed with 28 female players. Participants were divided into 2 groups, experimental and control, randomly: the EG (n=14, mean age 18.29, SD 0.99 y; mean height 174.50, SD 2.10 cm; mean weight 75.36, SD 2.10 kg; mean training 5.64, SD 0.50 y) and the CG (n=14, mean age 18.50, SD 0.65 y; mean height 175.64, SD 1.55 cm; mean weight 75.57, SD 1.79 kg; mean training 5.43, SD 0.51 y). The research inclusion criteria were established specifically to generate precise findings about the study. All participants needed to fulfill distinct qualifications, which included 6 female and active involvement in sports as well as healthy status, a minimum of 4 years of athletic experience, and a set age range. The study participants needed to exhibit continuous training participation until the end of the research period and successfully finish each assessment at the start and conclusion of the study. The study included fundamental exclusion criteria for eliminating factors that might produce confounding results during the research period. Athletes using medications or drugs were excluded because their substances could affect both their physiology and performance abilities. All participants who exhibited inconsistent training attendance or failed to perform baseline tests were automatically removed from the research. The established inclusion and exclusion criteria ensured homogeneity of the research sample as well as accurate data collection, which therefore strengthened the scientific rigor of this research.

### Ethical Considerations

The study did not need preinitiation registration. A clinical trial registry registration before initiation did not occur because this study falls outside the registered scope for clinical trials that mainly encompass drug or medical intervention studies. The study adhered to ethical guidelines and scientific standards for all procedures, from participant recruitment to randomization of participants and intervention delivery and data analysis, to maintain reliable results. All athletes received comprehensive knowledge about both risks and benefits that they would encounter during study participation before they joined. Each participant received an institutionally approved informed consent form while voluntarily signing it for written consent that established voluntary and uncoerced participation. The participants were not compensated or paid any monetary amounts since they volunteered to take part. The Research Ethics Committee at King Faisal University, Saudi Arabia, awarded ethical approval for the study protocol (approval KFU-REC-2024-MAR-ETHICS2088) (Multimedia Appendix 1). This study followed the international standards from the Declaration of Helsinki for protecting the rights, privacy, and welfare of human participants. The researchers took steps to ensure that participant data would stay confidential and serve only the research needs. Individuals who enrolled in the research received full disclosure about withdrawal rights as well as protection for their confidentiality as part of the procedure to safeguard their anonymity.

### Tools and Devices

**Figure 1.**
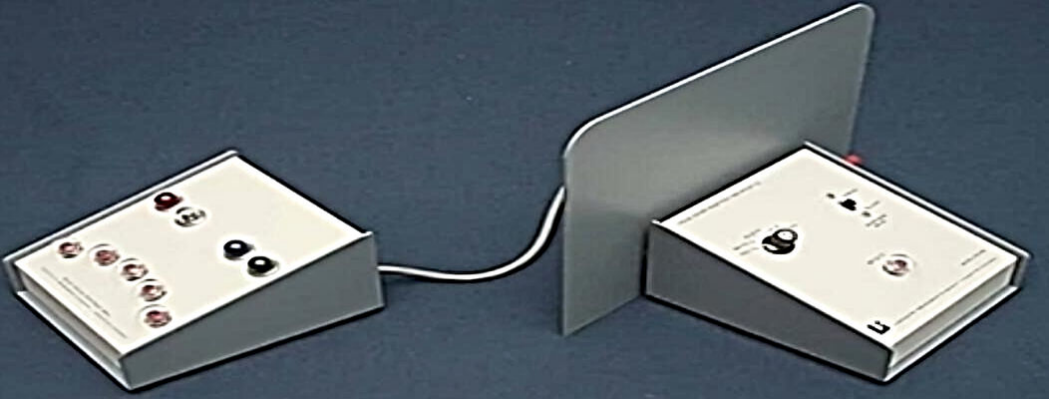
Visual reaction time apparatus 63035 (Multimedia Appendix 2).

**Figure 2.**
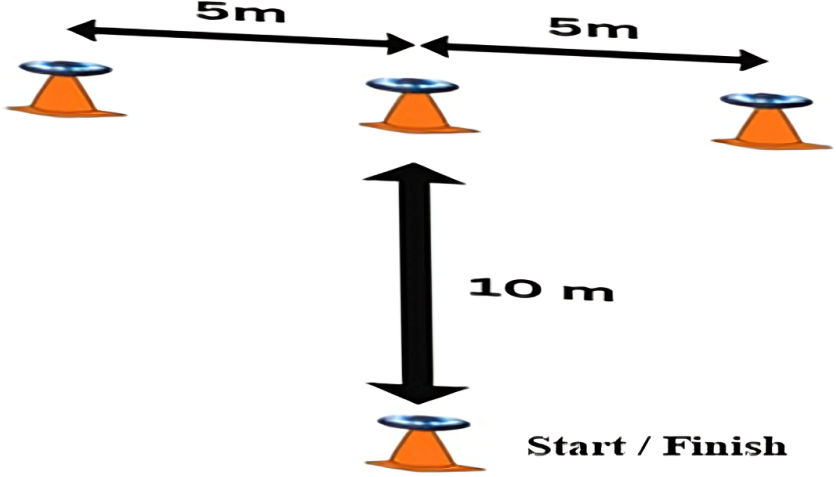
Modified *t* test with dribbling.

**Figure 3.**
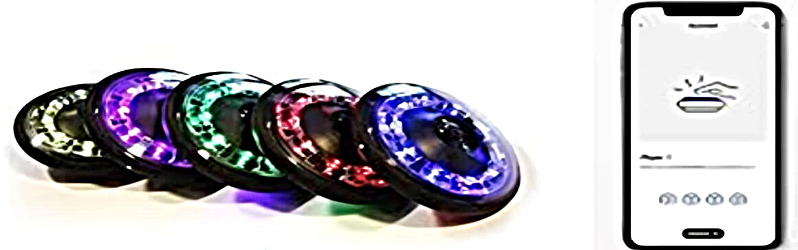
FITLIGHT technology.

The study incorporated various pieces of equipment for height and weight measurements, a restameter, a basketball, and the Device Response Panel 63035A combined reaction time (visual-motor interaction) obtained from the Lafayette Instrument Company (Figure 1) [9]. Furthermore, the modified *t* test was used to assess the speed of skillful dribbling (Figure 2) [11,24], and the FITLIGHT system (Figure 3), a stopwatch, and stands for the FITLIGHT disks and ropes were also used. The validity and reliability of the devices were further established via comparison with other instruments, such as the Visual Response Board 63035A (Lafayette Instrument Company) from another model exploding. To eliminate all doubts, these measurements were applied to additional participants in the current exploratory study. The Lafayette Instrument 63035A Reaction Time (visual-motor interaction) response board is illustrated in Figure 1; the instrument consists of 4 stimulus lamps, a Sonalert, and 5 response keys. The specifications are a line voltage of 105/125 V, AC/50/60 Hz power supply of 12 V DC from a wall-mounted transformer timer, a relay contact rating of 0.5 A/30 V DC and 0.5 A/50 V AC with an inductive load to be protected with arc suppression. The lamp used in the stimulus was 47/6.3 V Chicago Miniature [11]. Table 1 provides a summary of the training program (For further details, refer to MultimediaAppendices 2  3).

**Table 1.** Distribution of 10-week FITLIGHT exercise program for basketball players (total program time=4000 min).

Stage		General preparation			Special preparation				Precompetition preparation		
Week		1	2	3	4	5	6	7	8	9	10
Load^a^											
Maximum						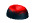		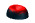	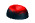		
High		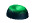	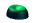		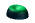						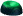
Medium				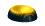			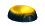			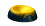	
Week time (min)		320	320	240	320	400	240	400	400	240	320

a The graphics represent training loads for maximum, high, and medium loads.

### Experimental Procedures

The researcher developed an exercise program using FITLIGHT technology that lasted for 10 weeks; there were 40 sessions in total, with 4 training sessions per week. The average duration of each session ranged from 60 to 100 minutes, of which the FITLIGHT exercises lasted for 25‐40 minutes. The level of exertion was checked and adjusted to guarantee an appropriate level of load that was varied and augmented in each phase of the program.

The EG followed these steps in each training unit:

Every session began with a set of generic warm-up exercises to prepare separate groups of muscles in the body. Different, specific warm-up exercises were then conducted to increase preparedness for the planned activities.

During the session, the researcher explained the nature of the exercises that would be performed using the FITLIGHT technique in relation to the objectives of the program.

Members of the EG completed the FITLIGHT exercises in the pre- and post–warm-up phases, in addition to the inter- and intramain session exercises to enhance visual-motor coordination and speed when dribbling. During the training, the researcher was always present for the EG and provided extra help, corrected mistakes, ensured that the participants were performing the exercises correctly, and ensured their safety. Finally, every session concluded with a cool-down stretching session to assist in recovery and subsequent flexibility.

In this program, the exercises were progressive in nature to ensure that the increase in the intensity and duration of the exercises was based on the improvements in the participants’ performances and their adaptation levels during the 10-week program (Table 1).

### Statistical Analysis

IBM-SPSS Statistics 26 served as the tool to conduct statistical analysis for achieving reliable results. The researchers performed descriptive statistical analysis to find minimum and maximum, mean, SD, variance, skewness, and coefficient of variation (CV) for the EG and CG. The CV measurement tool calculates group homogeneity by dividing the SD by mean values to determine percentages that show homogeneity, <30% indicating homogeneity and >30%. For analysis between and across groups, the inferential statistics involved paired *t* tests for measuring intragroup differences and independent *t* tests for evaluating performance distinctions between the EG and CG. The research calculated effect sizes through Cohen *d* and interpreted 3 effect magnitude levels, including small (<0.2), medium (0.2‐0.5), and large (>0.8). All statistical tests used a *P* value lower than .05 as the threshold to maintain consistency and reduce the concerns about multiple comparisons. Statistical computations of 95% CIs and mean differences, *t* values, and *P* values provided a deep analysis of the obtained results. The analysis followed standard conventions through these statistical methods, and the researchers applied these methods consistently during the entire analysis to ensure clear results and measurement reliability.

## Results

### Descriptive Statistics

**Figure 4.**
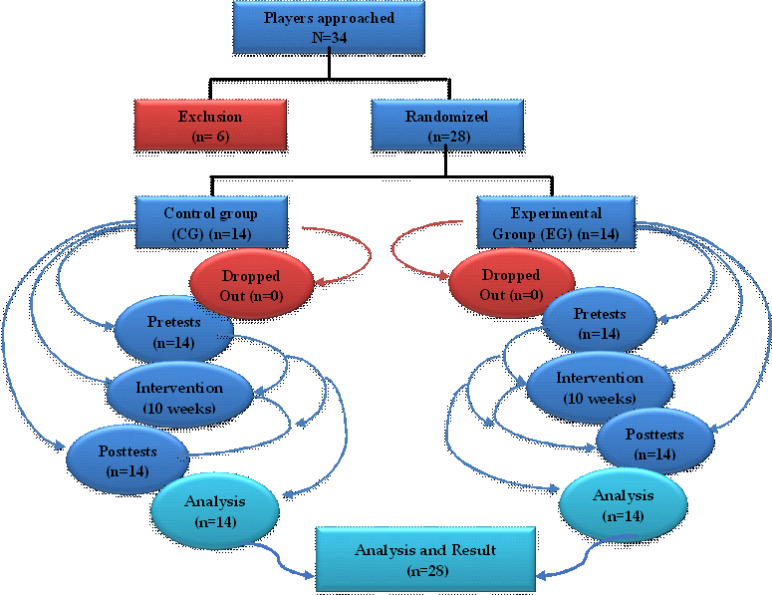
Flowchart in this study.

The flowchart of this study is illustrated in Figure 4. Analyzing the data in Table 2, an improvement in the homogeneity of performance within the EG compared with the CG during the tests was observed. Compared with the premeasurements, it is observable that the CV of the EG ranged from 0.63% to 5.41%. This reduction is indicative of increased reliability of the results that are generated, while the CG CV ranged from 0.39% to 2.26%, which can also be considered a good sign as it indicates good results consistency.

**Table 2.** Descriptive statistics for reaction tests and reactive tests.

Group and outcome measures	Test	Range, minimum-maximum	Mean (SD)	Variance	Skewness, statistic (SE)	CV^a^
EG^b^						
Age	Pre	17-20	18.29 (0.99)	0.99	−0.12 (0.60)	—^c^
Height	Pre	170-177	174.50 (2.10)	4.42	−1.42 (0.60)	—
Weight	Pre	70-77	75.36 (2.10)	4.40	−1.60 (0.60)	—
Training	Pre	5-6	5.64 (0.50)	0.25	−0.67 (0.60)	—
Reaction time in the right hand (ms)	Pre	0.401-0.420	0.41 (0.01)	0.00	0.65 (0.60)	2.44%
Reaction time in the right hand (ms)	Post	0.347-0.398	0.37 (0.02)	0.00	0.23 (0.60)	5.41%
Reaction time in the left hand (ms)	Pre	0.433-0.453	0.44 (0.01)	0.00	−0.18 (0.60)	2.27%
Reaction time in the left hand (ms)	Post	0.387-0.408	0.40 (0.01)	0.00	0.65 (0.60)	2.5%
Reactive time (sec)	Pre	12.57-12.88	12.78 (0.08)	0.01	−1.15 (0.60)	0.63%
Reactive time (sec)	Post	10.34-11.25	10.88 (0.32)	0.11	−0.35 (0.60)	2.94%
Reactive time with dribbling (s)	Pre	21.68-22.88	22.03 (0.43)	0.19	1.44 (0.60)	1.95%
Reactive time with dribbling (s)	Post	17.18-18.89	17.94 (0.45)	0.20	0.28 (0.60)	2.51%
CG^d^						
Age	Pre	18-20	18.50 (0.65)	0.42	0.98 (0.60)	—
Height	Pre	173-178	175.64 (1.55)	2.40	−0.17 (0.60)	—
Weight	Pre	72-77	75.57 (1.79)	3.19	−1.13 (0.60)	—
Training	Pre	5-6	5.43 (0.51)	0.26	0.32 (0.60)	—
Reaction time in the right hand (ms)	Pre	0.401-0.420	0.410 (0.006)	0.00	−0.34 (0.60)	1.46%
Reaction time in the right hand (ms)	Post	0.382-0.401	0.394 (0.004)	0.00	−1.15 (0.60)	1.02%
Reaction time in the left hand (ms)	Pre	0.433-0.453	0.444 (0.007)	0.00	−0.18 (0.60)	1.58%
Reaction time in the left hand (ms)	Post	0.401-0.445	0.425 (0.013)	0.00	−0.29 (0.60)	3.06%
Reactive time (s)	Pre	12.68-12.88	12.83 (0.05)	0.00	−1.76 (0.60)	0.39%
Reactive time (s)	Post	11.73-12.21	11.98 (0.14)	0.02	−0.02 (0.60)	1.17%
Reactive time with dribbling (s)	Pre	21.76-22.88	22.05 (0.43)	0.18	1.48 (0.60)	1.95%
Reactive time with dribbling (s)	Post	19.84-21.45	20.34 (0.46)	0.21	0.91 (0.60)	2.26%

a CV: coefficient of variation.

b EG: experimental group.

c Not applicable.

d CG: control group.

### Paired Sample *t* Test

Table 3 shows the results of the assessment. The statistical analysis of the obtained scores indicates significant superiority of the EG over the CG for all the assessments carried out. Significant improvements were observed in the EG, as the differences in performance pre- and postmeasurements were higher (*P*<.001). The Cohen *d* of all the tests was significantly higher for the EG, ranging between 0.87 and 0.98. This means that the group conducted an experiment and made a significant level of achievements, as shown by the size of the effect. Specifically, the CG had a comparatively lower magnitude of change in the outcome variables. The effect size for the CG varied from 0.71 to 0.97, which is much lower than for the EG. The steady increase in the difference of improvement between the EG and the CG adds credence to the training program conducted for the EG.

**Table 3.** Statistical analysis of paired sample *t* test for reaction time and reactive time.

Outcome measures and group	Test	Mean difference (SD)	SE	95% CI	*t* test (df)	*P* value	Cohen *d*^a^
Reaction time in the right hand (ms)							
EG^b^	Pre-post	0.04 (0.02)	0.00	0.03-0.05	9.22 (13)	<.001	0.87
CG^c^	Pre-post	0.016 (0.009)	0.002	0.01-0.021	7.10 (13)	<.001	0.79
Reaction time in the left hand (ms)							
EG	Pre-post	0.05 (0.01)	0.00	0.04-0.05	19.07 (13)	<.001	0.97
CG	Pre-post	0.019 (0.012)	0.003	0.012-0.03	5.71 (13)	<.001	0.71
Reactive time (s)							
EG	Pre-post	1.90 (0.31)	0.08	1.72-2.08	22.70 (13)	<.001	0.98
CG	Pre-post	0.85 (0.15)	0.04	0.76-0.94	20.52 (13)	<.001	0.97
Reactive time with dribbling (s)							
EG	Pre-post	4.08 (0.67)	0.18	3.70-4.47	22.85 (13)	<.001	0.98
CG	Pre-post	1.71 (0.49)	0.13	1.43-1.99	13.04 (13)	<.001	0.93

a d: effect size.

b EG: experimental group.

c CG: control group.

### Independent *t* Test

In the analysis of the data presented in Table 4, while comparing the performance of the 2 groups, the premeasurements suggest that both the EG as well as the CG offered similar performance in all the tests conducted. This similarity at the start of the study means there are no differences in the visual-motor interaction and dribbling speed between the 2 groups that would give one an unreasonable edge over the other. On the other hand, the post measures showed significant differences in the performance of the EG in comparison with the CG. This improvement is evidenced by considerably better performance in all the assessed tests. Cohen *d* is ranging between 0.51 and 0.88. The probability values post measurements work as pointers toward a fair and highly significant improvement that is not arbitrary and that is attributed to the revelation of a structured training program intended for the improvement of visual-motor coordination and dribbling speed among the female basketball players.

**Table 4.** Statistical analysis of independent *t* test for reaction time and reactive time for experimental group and control group.

Outcome measures and test		Levene test		*t* test		Mean difference (SE)		95% CI		Cohen *d*
		*F* test (df)	*P* value	*t* test (df)	*P* value					
Reaction time in the right hand (ms)										
Pre		0.14 (26)	.71	1.18 (26)	.25	0.00 (0.00)		−0.01 to 0.00		0.05
Post		26.49 (26)	<.001	5.19 (26)	<.001	−0.02 (0.00)		−0.03 to −0.01		0.51
Reaction time in the left hand (ms)										
Pre		0.00 (26)	>.99	0.00 (26)	>.99	0.00 (0.00)		−0.01 to 0.01		0.00
Post		11.41 (26)	<.001	7.91 (26)	<.001	−0.03 (0.00)		−0.04 to −0.02		0.71
Reactive time (s)										
Pre		2.25 (26)	.15	1.89 (26)	.07	−0.05 (0.03)		−0.10 to 0.00		0.12
Post		13.32 (26)	<.001	11.66 (26)	<.001	−1.10 (0.09)		−1.29 to −0.91		0.84
Reactive time with dribbling (s)										
Pre		0.01 (26)	.93	0.15 (26)	.88	−0.02 (0.16)		−0.36 to 0.31		0.00
Post		0.002 (26)	.97	13.88 (26)	<.001	−2.39 (0.17)		−2.75 to −2.04		0.88

## Discussion

### Principal Findings

The purpose of this study was to evaluate the effectiveness of a basketball training program involving the use of FITLIGHT technology on female basketball players’ visual-motor coordination and dribbling speeds. A comparative analysis of the results of each of the tests indicated the positive impact of the experimental training program on the aspects of visual-motor coordination and dribbling. When evaluating the results obtained before and after the implementation of the program for the EG and CG, statistically meaningful changes throughout the assessments in this research were evident. The EG’s achievements in all the assessments were not only better but were statistically greater than the CG achievements. In addition, higher Cohen *d* values for the EG supported and highlighted not only the basic statistical evidence of the treatment’s effect but also quantified the impact of the FITLIGHT training program on the improvements in the visual-motor coordination levels and dribbling speeds of the female basketball players.

The study has demonstrated that FITLIGHT exercises enhance visual-motor coordination along with dribbling speeds, primarily because they engage light stimuli that improve motor skills and reaction times. The specific brain centers that process visual information and control motor responses become activated because of this enhancement. FITLIGHT technology demonstrates the successful development of reactive coordination and movement combination capacity in basketball players since EGs showed more notable gains than CGs [24]. The application of light-based training methods in soccer enhances visual and visuomotor capabilities through improved performance in dynamic visual acuity and sensory reaction time, according to the findings of Rodrigues et al [25]. Small-sided games that incorporate FITLIGHT training have proven effective for improving basketball performance at both harmonic abilities and basic skills to benefit a wide range of sports training [9]. FITLIGHT, during reactive agility training, produced noteworthy improvements in both visual reaction times and dribbling skills because of the system’s success in athlete performance strengthening [11].

The visual cortex processing of visual information is enhanced for athletes following their engagement with FITLIGHT exercises because these workouts activate the visual cortex. The available research demonstrates that reaction times, together with cognitive functionality and motor skills, improve after such training becomes part of athletes’ preparation programs across various sports like basketball, soccer, and tennis. A visual stimuli program led young soccer players to achieve an 11.8% enhancement of their reaction times alongside important cognitive improvement, according to research [26]. Tennis players who belonged to the 10-year-old category showed advancements in both reaction times and motor performance after using FITLIGHT technology for their visual training [15]. The research shows that athletic performance benefits from FITLIGHT exercises that activate the visual cortex [27].

Neuroplasticity enables significant neural efficiency enhancement through repetitive FITLIGHT training because it allows the brain to restructure and reinforce neural connections as a result of learning and exposure. Research shows that such educational programs enhance cognitive-motor abilities by improving athletes’ reactive coordination, combined with better reaction times and visual-motor responses. The reactive abilities and dribbling skills of basketball players substantially improved after FITLIGHT training based on effect sizes that showed a large impact [11,24,28]. Judo athletes achieved better executive functions and motor performance results after completing FITLIGHT cognitive-motor training because the technology helps brains adapt sensory-motor integrative mechanisms [7,27].

The cerebellum functions to refine motor control for precise movements, especially when athletes need quick responses to visual cues when dribbling basketballs. Scientific evidence shows that the cerebellum has exceptional precision when encoding dynamic motor frequencies to produce uniform motor kinematics across all people needed for accurate and timed activities [29-32]. When athletes take part in FITLIGHT sessions, their improved responses allow the cerebellum, together with the cerebral cortex, to adapt action precision and timing that result in better athletic performance [31]. The cerebellum plays additional tasks across motor control by affecting cognitive and emotional processes, leading to improved athlete performance during pressure situations [33]. The cerebellum plays a key role in complex motor tasks, together with its role in sports performance [34].

While the FITLIGHT system is not age-specific, it is used as a tool for determining response times, attention, concentration, physical activity, and overall fitness. The training program itself was developed to enhance reactive ability, hand-eye coordination, and the ability to perform movements in a direct and highly intensive manner using the FITLIGHT technologies. The application of these technologies in the framework of the experimental program was time-sensitive, which enabled the further evolution of the training methodology depending on the outcomes that the athletes achieved. As a result, the players improved their reaction to stimuli, and their control of movement patterns in basketball-specific training situations had higher accuracy and coordination. The results of our study are confirmed by previous studies [9,11,12,19,35-38]. The physical activity and the quality of a sport, together with the developmental progress of a player, are related to the improvements in response times that were also identified in this study. Reaction time plays an important role in different game conditions in which the athlete should make the correct decision in the shortest time to guarantee the successful execution of an action. Hence, improvements in response times are correlated positively with the quality and development of physical activity and sports.

Several papers have compared the effectiveness of basketball players’ training and changes in training programs using FITLIGHT technology [24,39]. When incorporating visual stimuli using different techniques and parallel types of stimulation via touch and movement, the result was a positive increase in the athletic abilities that are demanded in sports disciplines [40,41]. Badau et al [42] integrated 3 months of sports training using FITLIGHT technology into open-skill sports for adolescents, such as basketball, handball, and volleyball. This led to the athletes having significantly faster cognitive reaction rates, which are a measure of cognitive flexibility [42]. The study by Silvestri et al [27] found that the use of FITLIGHT technology in training enhanced the executive functions without reducing the enjoyment aspect of training sessions. A separate study, which involved 154 male and female volleyball and basketball players [43], also revealed that using FITLIGHT technology helped to enhance the athletes’ interactions.

Thus, this work substantially enriches the existing knowledge about the application of FITLIGHT technology to optimize visual-motor coordination and the dribbling speeds of female basketball players. Coordination and rapid reaction capabilities are crucial components in the training of basketball athletes; these abilities can be improved using FITLIGHT in a way that is compatible with the challenges that basketball players encounter. Designing a specific program based on the use of information technologies that concern visual-motor coordination and response rates can significantly enhance athletic abilities. According to FITLIGHT, this allows athletes to enhance their coordination and responsiveness in a setting that mimics real-time basketball training. Furthermore, it enhances motivation for training and ensures the achievement of mastery in the technical aspects of basketball. With practice in the visual-motor skills and the reactions that are needed in training being customized, athletes can complete complicated actions and then make sound, rapid decisions in sports. Therefore, our results support the feasibility of applying sophisticated training technologies to boost or elevate sports performance and overall basketball training.

### Strength

The study benefits from its usage of FITLIGHT technology to introduce a modern method for enhancing both visual-motor coordination and dribbling speed abilities. The research design used controlled experiments to study the FITLIGHT intervention through comparisons between the EG and CG. Analysis methods containing complete statistical methods provided reliable findings across the 10-week training cycle. Near-instant data collection tools measured reaction and reactive times, which showed substantial improvements in the EG results. The research results indicate that structured training approaches effectively boost athlete competency alongside performance reliability for coaches to consider. The established training method minimizes performance alterations while helping female basketball players improve their skills.

### Limitation

#### Sample Size

The research included just 28 participants, whereby 14 participants received FITLIGHT training in the EG while 14 participants were allocated as the CG. The study’s restricted participant population reduces the ability of the results to extend to different female athletes in society. A bigger participant number, exceeding the current 28, would help increase the quality of study findings and deliver a more detailed view of FITLIGHT training effects.

#### Duration of the Training Program

The 10-week duration of training seemed inadequate to determine how FITLIGHT affects visual-motor coordination and ball control speed exclusively. The training program results could show different outcomes with prolonged training duration at each session.

#### Nature of the Sporting Context

The authors applied their research exclusively to basketball because the study investigated this sport specifically. The findings cannot extend to sports that require dissimilar visual-motor abilities and different skill requirements.

### Recommendations and Future Studies

The investigation shows that training techniques incorporating FITLIGHT systems should be integrated into programs for female basketball athletes because this technology helps develop their visual-motor coordination while speeding up ball handling. FITLIGHT allows players to receive personalized training plans from coaches who use this system to achieve balanced management of team and individual sessions. An extension of visual response time and motor coordination training will boost total player performance by strengthening the connection between the brain and muscles while minimizing athletic performance inconsistencies. Training and developing institutions should create instructions for FITLIGHT use and selection of advanced fitness technologies, which will enable coaches to enhance their performance results. The study will gain increased validity if future research analyzes more female basketball players through the same methodology while including additional comparative evaluation of male and female performance. The assessment should analyze how anthropometric markers (height and weight) affect performance results following a FITLIGHT implementation, along with evaluating extended training procedures (over 10 wk) to determine their effect on athletic skill advancement. The long-term effects of this technology on athletic performance and female athlete mental health need evaluation, while researchers should compare it with established visual-motor coordination improvement techniques. An investigation of how FITLIGHT training affects mental resilience and mental focus development during competitions needs further evaluation. Research involving this technology can establish a complete assessment of its performance-enhancing impact on athletic prowess and mental well-being.

### Conclusions

The study investigated the power of FITLIGHT technology-based basketball training methods as they enhanced visual-motor coordination capabilities and ball control speed for female basketball athletes. The 10-week duration of the program generated statistical differences between those participating in the EG compared with the CG participants. The EG enhancements exceeded those of the CG because research participants achieved CV improvements between 0.63% and 5.41% during the intervention period, but the CG CV stayed in the 0.39% to 2.26% range. Data analysis through paired sample *t* tests demonstrated that the EG surpassed the CG in all assessments, while obtaining large effect sizes between 0.87 and 0.98 for the EG compared with 0.71 to 0.97 for the control CG. Independent *t* test analysis confirmed no preintervention differences, yet the intervention led to substantial and statistically important improvements for both groups, and Cohen *d* values spanned between 0.51 and 0.88. The training program specifically improves visual response time, together with visual-motor coordination capabilities. The obtained data support implementing systematic training programs anchored in modern methods, including FITLIGHT, for minimizing performance variability and enhancing skill consistency.

## Supplementary material

10.2196/70519 Multimedia Appendix 1 Ethical clearance.

10.2196/70519 Multimedia Appendix 2 Materials used for the visual-motor interaction test.

10.2196/70519 Multimedia Appendix 3 Training program for exercises using the Fitlight system.

## Abbreviations

CG: control group
CV: coefficient of variation
EG: experimental group
