# Correction: FITLIGHT Training and Its Influence on Visual-Motor Reactions and Dribbling Speed in Female Basketball Players: Prospective Evaluation Study

**DOI:** 10.2196/82614

**Published:** 2025-09-12

**Authors:** Ahmad K Hassan

**Affiliations:** 1 Department of Physical Education, College of Education, King Faisal University, P.O. Box 400, Al-Ahsa, 31982, Saudi Arabia, 966 536980427; 2 Department of Team Sports and Racket Games, Faculty of Physical Education, Minia University, Minia, Egypt

In “FITLIGHT Training and Its Influence on Visual-Motor Reactions and Dribbling Speed in Female Basketball Players: Prospective Evaluation Study” (JMIR Serious Games 2025;13:e70519), the author noted two corrections.

The corresponding email address was changed to “amohhamed@kfu.edu.sa.”

In addition, the following affiliation was added: “Department of Team Sports and Racket Games, Faculty of Physical Education, Minia University, Minya, Egypt.”

The correction will appear in the online version of the paper on the JMIR Publications website, together with the publication of this correction notice. Because this was made after submission to PubMed, PubMed Central, and other full-text repositories, the corrected article has also been resubmitted to those repositories.

