# Supplementary material for: FITLIGHT Training and Its Influence on Visual-Motor Reactions and Dribbling Speed in Female Basketball Players: Prospective Evaluation Study
Source: JMIR Serious Games. 2025 Jul 4;13:e70519. doi: 10.2196/70519 (PMC12252139; doi:10.2196/70519)
Supplement: Multimedia Appendix 1 [file games-v13-e70519-s001.pdf]

Deanship of Scientific Research

موافقة أخلاقية
Ethical Clearance

Ref. No.	KFU-REC-2024-MAR-ETHICS2088	الرقم المرجعي
Project Title	FITLIGHT Training and Its Influence on Visual-Motor Reactions and Dribbling Speed in Female Basketball Players	اسم المشروع البحثي
Contact Point	Ahmed Khalifa Hassan	ضابط الاتصال
Phone	+966536980427	الهاتف
Email	amohhamed@kfu.edu.sa	البريد الإلكتروني
Co-Researcher 1	Ahmed Khalifa Hassan	باحث 1
Granting Institution	جامعة الملك فيصل<br>King Faisal University	المؤسسة المانحة
Approval Date	26/ 03 /2024	تاريخ الموافقة
Approval Validity	24 Months	صلاحية الموافقة

تفيد لجنة أخلاقيات البحث العلمي بجامعة الملك فيصل بأنه تم منح المشروع البحثي الموضح عاليه موافقة أخلاقيات البحث العلمي، وذلك بناء على فحص للجانب الأخلاقي من المشروع وفقاً للبيانات المزودة من قبل صاحب الطلب. قد تخضع المشاريع البحثية للمتابعة الميدانية أو أي شكل آخر من أشكال المتابعة الدقيقة من قبل اللجنة في أي وقت. قد تطلب اللجنة تقريرًا منتظمًا عن تقدم المشروع لضمان التزام الباحثين بأعلى المعايير الأخلاقية. الباحثون مسؤولون عن تخزين وحفظ وتأمين البيانات الناجمة عن المشاريع. يجب على الباحثين إبلاغ اللجنة على الفور بأي تعديلات جوهرية على المشروع أو قضايا أخلاقية مستجدة وذلك من خلال البريد الإلكتروني (ialjreesh@kfu.edu.sa) أو الهاتف (٠٠٩٦٦١٥٨٩٩٧٧٣).

Having reviewed the details submitted by the applicant regarding the abovenamed research project, the Research Ethics Committee at King Faisal University grants its ethical approval to the protocol. Projects may be subject to an audit or any other form of monitoring by the committee at any time. The committee may request a regular report on the progress of the project to ensure that researchers are committed to the highest ethical standards. Researchers are held accountable for the storage, retention and security of original data obtained from projects. Any substantial alterations to the project or emerging events or matters that may affect the ethical acceptability of the project must be reported immediately to the committee via email (ialjreesh@kfu.edu.sa) or phone (0096615899773).

The Chair of the Research Ethics Committee	د.منصور بن عبدالله اليحيا<br>Dr.Mansour Abdullah Alyahya	رئيس لجنة أخلاقيات البحث العلمي
Date	26/03/2024	التاريخ

عمادة البحث العلمي
SCIENTIFIC RESEARCH DEANSHIP

Informed Consent

الموافقة على التبصير

You are invited by (Dr. Ahmed khalefa Hassan) to participate in a scientific research.

أنـت مـدعو(ة) مـن قبـل (د. أحمد خليفه حسن) إلى المشاركة في بحث علمي.

Research Title	FITLIGHT Training and Its Influence on Visual-Motor Reactions and Dribbling Speed in Female Basketball	عنوان البحث
Ethical Committee Approving the Research	King Faisal University's Committee<br>اللجنة الخاصة بجامعة الملك فيصل	اسم المنشأة التي اعتمدت البحث
Research Objectives	impact of a training program implementing FITLIGHT technology on female basketball players' visual-motor interactions and dribbling speed.	أهداف البحث
Expected Outcomes or Benefits for the Person under Study	None	المنفعة المتوقعة للشخص موضع البحث
Expected Risks or Harms to the Person under Study	None	الخطر المحتمل للشخص موضع البحث أو المجتمع وكذلك الخطر الذي يمكن أن يترتب على الانسحاب من البحث
Procedures or Medical Treatments Related to the Study	N/A	طرق العلاج البديلة المتوافرة خارج نطق البحث
Alternative Treatments Available outside the Scope of the Study	N/A	الإجراءات (والمعالجات الطبية) التابعة للبحث
Study Duration	10 weeks	مدة إجراء البحث
Requirements from the Person under Study	Join the training program	المطلوب من الشخص موضع البحث
Type and Quantity of Samples Taken from the Person under Study and how It will Be Used	N/A	نوع العينات التي ستؤخذ من الشخص موضع البحث وكميتها وكيفية استخدامها
How the Person under Study Is Compensated if s/he Sustains any Harm Resulting from the Study	N/A	كيفية تعويض الشخص الذي سيجرى عليه البحث في حال وقع ضرر ناتج عن إجراء البحث عليه

The person under study can use the following contact information to obtain further information related to the study or to report any harm sustained:

للشـخص موضـع البحـث التواصـل مـع أرقـام وعنـاوين الاتصـال التاليـة للحصـول علـى أيـة معلومـات تتعلـق بالبحث أو بحقوقه أو التبليغ في حالة إصابته بضرر

Email of the Ethical Committee at King Faisal University	ialjreesh@kfu.edu.sa	البريد الإلكتروني للجنة أخلاقيات البحث العلمي بجامعة الملك فيصل
WhatsApp of the Ethical Committee at King Faisal University	0096615899773	واتساب لجنة أخلاقيات البحث العلمي بجامعة الملك فيصل
Email of Lead Researcher	amohhamed@kfu.edu.sa	البريد الإلكتروني للباحث الرئيس
Mobile Phone of Lead Researcher	0536980427	رقم جوال الباحث الرئيس

- The researcher confirms the confidentiality of information that could reveal the identity of the human subject.
- Participation in the research is voluntary. Refusal to participate shall not entail a loss of benefits to which the human subject would otherwise be entitled. The human subject may withdraw from the research at any phase without loss of benefits to which he is otherwise entitled.
- The Principal Investigator's pledge that the human subject shall be notified of all information that may emerge during the research period, the knowledge of which may affect his decision for continued participation in the research.

- يقر الباحث بالالتزام بالمحافظة على سرية المعلومات التي يمكن أن تؤدي إلى تحديد هوية الشخص الذي سيجرى عليه البحث.
- المشاركة في البحث أمر طوعي، ورفض المشاركة لن يترتب عليه أية خسارة لمنفعة يستحقها الشخص موضع البحث، وللشخص موضع البحث الحق في الانسحاب من البحث في أية مرحلة من مراحله دون أن يتعرض لخسارة أو فوات منفعة يستحقها لأي سبب.
- يتعهد الباحث بأن الشخص موضع البحث سيحاط علماً بجميع المعلومات التي قد تستجد خلال مدة إجراء البحث والتي يمكن أن تؤثر معرفته بها في استمرار مشاركته في البحث.

Name of the Person under Study or his/her Guardian		اسم الشخص الذي سيجرى عليه البحث أووليه
Signature of the Person under Study or his/her Guardian		توقيع الشخص الذي سيجرى عليه البحث أووليه
Name of the Lead Researcher	Ahmed khalifa hassan	اسم الباحث الرئيس
Signature of the Lead Researcher	dr ahmed khalifa hassan	توقيع الباحث الرئيس
Date of the Consent		تاريخ الموافقة على المشاركة
