# Supplementary material for: FITLIGHT Training and Its Influence on Visual-Motor Reactions and Dribbling Speed in Female Basketball Players: Prospective Evaluation Study
Source: JMIR Serious Games. 2025 Jul 4;13:e70519. doi: 10.2196/70519 (PMC12252139; doi:10.2196/70519)
Supplement: Multimedia Appendix 2 [file games-v13-e70519-s002.docx]

**(Appendix A):**

**visual-motor Interaction test**

This accessory contains an Attention Focus measurement device “Li Lafayette Instrument Visual Reaction Time Apparatus 63035A Response Panel” (Figures1)

This unit is versatile for use with an external timer to measure both simple and discriminate reaction times. Complete with four stimulus lamps, a Sonalert, for auditory stimulation, and five response keys,this unit is used to provide simultaneous contact closure with stimulus onset and simultaneous contact release with the correct response for accurate measurement of reaction times. Although any timer can be used with this basic unit, Line Voltage (Model 63035) 105/125V AC 50/60 Hz, Power (Model 63035A) 12V DC Wall mount transformer, Timer Relay Contact Rating 0.5 amps @ 30V DC, 0.5 amps @ 50V AC, Inductive loads should be arc Supressed, Stimulus Lamps #47 6.3V Chicago-Miniature

**Operation Instructions:**

The control panel plugs into any suitable AC outlet (Model 63035A only). With the Model 63035A, the 12-volt wall-mount adapter plugs into an AC outlet and also plugs into the power receptacle on the side of the control box, connecting the meter to the tie-down posts provided on the control panel. All recommended timers have their own internal power supplies and can be connected directly to the control board. The stimulus (visual - auditory) is selected and the start button is pressed. This will activate the selected stimulus and timer. The response mode selector switch determines the subject's necessary response action to the stimulus. In DEPRESS mode, the response should be triggered by pressing the appropriate response key after the desired stimulus has been identified and initiated. In RELEASE mode, the selected response key must be pressed when the stimulus is initiated, and respond by releasing the key. When the correct response is made, the stimulus will be extinguished and the timer deactivated. Please note that only the middle (third) key will disable the auditory stimulus.


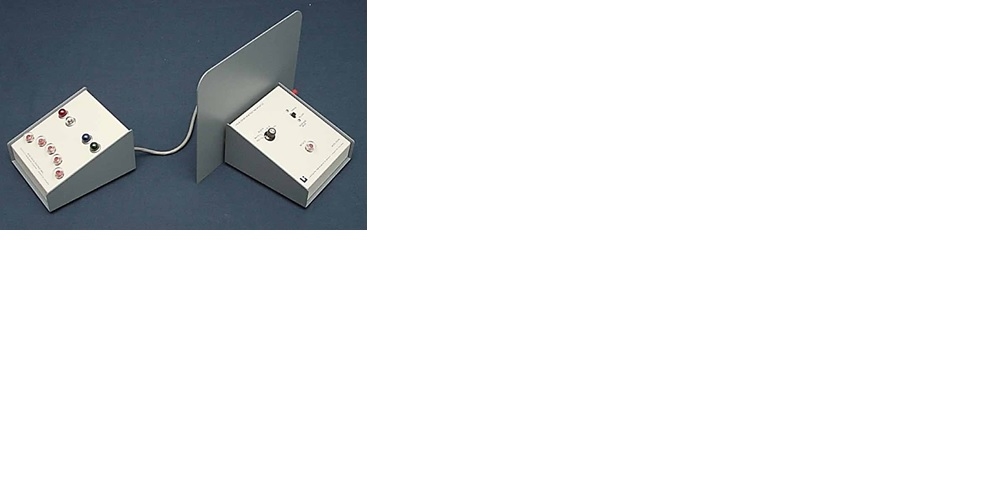


Reactive Agility T-Test (RAT) with Dribbling and Shooting: This test was conducted with the assistance of the Fitlight system placed on cones as depicted below in figure 3. In the experiments, the athletes were required to perform movements in response to lights signals and switching from one direction to another with dribbling. They also changed the movements swiftly.At the end of the test, the athletes shooting. In this version of the Reactive Agility T-Test, agility and the participants’ reaction time were measured depending on their response to the information indicated within some time limit. The Fitlight rule was arranged in such a way that either of the two dots placed at the end of the T was bright when the middle dot was touched randomly [1,9,34]. The time spent by the participant was summed up as each participle was allowed to try the activity three times and only the result of the best trial was taken as the measure for dribbling time While shooting time was calculated in the following manner If the ball hit the basket is counted as two points if the ball touched the board is counted as one point and if the ball not touched is counted as zero Each of the experimenters was allowed to three attempt the activity and the average was calculated.
