# Supplementary material for: FITLIGHT Training and Its Influence on Visual-Motor Reactions and Dribbling Speed in Female Basketball Players: Prospective Evaluation Study
Source: JMIR Serious Games. 2025 Jul 4;13:e70519. doi: 10.2196/70519 (PMC12252139; doi:10.2196/70519)
Supplement: Multimedia Appendix 3 [file games-v13-e70519-s003.docx]

**Appendix 3:**

S1. 10-week FITLIGHT exercise program for basketball players.

| Stage | | General preparation | | | Special preparation | | | | Pre-competition preparation | | |
| --- | --- | --- | --- | --- | --- | --- | --- | --- | --- | --- | --- |
| Week | | 1 | 2 | 3 | 4 | 5 | 6 | 7 | 8 | 9 | 10 |
| Load | **Max** |  |  |  |  | 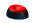 |  | 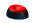 | 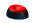 |  |  |
|  | **High** | 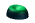 | 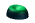 |  | 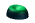 |  |  |  |  |  | 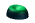 |
|  | **Mid** |  |  | 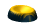 |  |  | 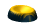 |  |  | 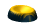 |  |
| Week Time | | 320 min | 320 min | 240 min | 320 min | 400 min | 240 min | 400 min | 400 min | 240 min | 320 min |
| Total Program Time | | 4000 min | | | | | | | | | |

S2. The Content of FITLIGHT Exercises

| **Weeks** | **Week 1** | | | | **Week 2** | | | | **Week 3** | | | | **Week 4** | | | | **Week 5** | | | | **Week 6** | | | | **Week 7** | | | | **Week 8** | | | | **Week 9** | | | | **Week 10** | | | |
| --- | --- | --- | --- | --- | --- | --- | --- | --- | --- | --- | --- | --- | --- | --- | --- | --- | --- | --- | --- | --- | --- | --- | --- | --- | --- | --- | --- | --- | --- | --- | --- | --- | --- | --- | --- | --- | --- | --- | --- | --- |
| **Severity** | **60%** | | | | **65%** | | | | **65%** | | | | **70%** | | | | **70%** | | | | **75%** | | | | **75%** | | | | **80%** | | | | **85%** | | | | **85%** | | | |
| **Days** | 1 | 2 | 3 | 4 | 5 | 6 | 7 | 8 | 9 | 10 | 11 | 12 | 13 | 14 | 15 | 16 | 17 | 18 | 19 | 20 | 21 | 22 | 23 | 24 | 25 | 26 | 27 | 28 | 29 | 30 | 31 | 32 | 33 | 34 | 35 | 36 | 37 | 38 | 39 | 40 |
| **Warm Up** | **Exercises to prepare the body and extensions** | | | | | | | | | | | | | | | | | | | | | | | | | | | | | | | | | | | | | | | |
| **FitLight Exercises** | **1** | **7** | **4** | **3** | **5** | **13** | **6** | **3** | **9** | **1** | **13** | **7** | **13** | **5** | **15** | **11** | **3** | **9** | **15** | **13** | **7** | **13** | **15** | **15** | **11** | **3** | **15** | **9** | **1** | **15** | **5** | **7** | **14** | **13** | **15** | **12** | **10** | **15** | **12** | **14** |
|  | **2** | **8** | **6** | **4** | **6** | **14** | **8** | **4** | **10** | **2** | **14** | **8** | **14** | **6** | **16** | **12** | **16** | **10** | **16** | **14** | **8** | **14** | **16** | **6** | **12** | **4** | **16** | **10** | **2** | **16** | **7** | **8** | **15** | **14** | **18** | **13** | **11** | **16** | **15** | **15** |
|  | **3** | **9** | **8** | **5** | **7** | **1** | **9** | **5** | **11** | **3** | **1** | **9** | **1** | **7** | **17** | **13** | **17** | **13** | **17** | **1** | **10** | **1** | **17** | **12** | **13** | **5** | **17** | **11** | **3** | **17** | **11** | **9** | **17** | **17** | **19** | **15** | **13** | **17** | **17** | **16** |
|  | **4** | **10** | **11** | **6** | **8** | **2** | **10** | **6** | **12** | **4** | **2** | **10** | **2** | **8** | **18** | **14** | **18** | **15** | **18** | **2** | **11** | **2** | **18** | **8** | **14** | **6** | **18** | **12** | **4** | **18** | **13** | **10** | **19** | **18** | **20** | **17** | **15** | **18** | **19** | **18** |
|  | **5** | **11** | **12** | **7** | **9** | **3** | **11** | **7** | **13** | **5** | **3** | **11** | **3** | **9** | **19** | **1** | **20** | **16** | **19** | **3** | **12** | **3** | **20** | **9** | **1** | **7** | **19** | **13** | **5** | **19** | **15** | **20** | **20** | **19** | **12** | **18** | **16** | **19** | **20** | **20** |
| **Group** | **2** | **3** | **3** | **3** | **3** | **3** | **3** | **2** | **3** | **3** | **2** | **3** | **3** | **3** | **2** | **3** | **3** | **3** | **2** | **3** | **3** | **3** | **2** | **3** | **3** | **3** | **2** | **3** | **3** | **3** | **2** | **3** | **3** | **3** | **3** | **2** | **3** | **3** | **3** | **2** |
| **finally** | **recovery exercises** | | | | | | | | | | | | | | | | | | | | | | | | | | | | | | | | | | | | | | | |

**FITLIGHT Exersise**

1. **Jumping Jacks**
   - **Repetitions: 15-20**
   - **Sets: 3**
2. **Push-Ups**
   - **Repetitions: 10-15**
   - **Sets: 3**
3. **Squats**
   - **Repetitions: 15-20**
   - **Sets: 3**
4. **Lunges**
   - **Repetitions: 10-12 per leg**
   - **Sets: 3**
5. **Leg Raises**
   - **Repetitions: 12-15**
   - **Sets: 3**
6. **Plank**
   - **Duration: 30-60 seconds**
   - **Sets: 3**
7. **Pull-Ups**
   - **Repetitions: 5-10**
   - **Sets: 3**
8. **Shoulder Press**
   - **Repetitions: 10-12**
   - **Sets: 3**
9. **Running in Place**
   - **Duration: 1-2 minutes**
   - **Sets: 3**
10. **Jump Rope**
    - **Duration: 1-2 minutes**
    - **Sets: 3**
11. **Dumbbell Curls**
    - **Repetitions: 10-12**
    - **Sets: 3**
12. **Chest Press**
    - **Repetitions: 10-12**
    - **Sets: 3**
13. **Sit-Ups**
    - **Repetitions: 15-20**
    - **Sets: 3**
14. **Side Stretch**
    - **Duration: 30 seconds per side**
    - **Sets: 2**
15. **High Knees**
    - **Duration: 30-60 seconds**
    - **Sets: 3**
16. **Single Leg Deadlift**
    - **Repetitions: 10-12 per leg**
    - **Sets: 3**
17. **Back Extensions**
    - **Repetitions: 12-15**
    - **Sets: 3**
18. **Balance Exercise**
    - **Duration: 30-60 seconds per leg**
    - **Sets: 2**
19. **Box Jumps**
    - **Repetitions: 8-10**
    - **Sets: 3**
20. **Cool Down Stretch**
    - **Duration: 5-10 minutes**
    - **Sets: 1**

**Warm-Up Exercises with FITLIGHT**

| **Exercise** | **Repetitions / Duration** | **Sets** | **FITLIGHT Integration** |
| --- | --- | --- | --- |
| **Jumping Jacks** | **15-20 reps** | **3** | Place lights on the floor or walls. Activate lights randomly; jump to touch each light. |
| **Running in Place** | **1-2 minutes** | **3** | Set lights in front of you. React to light cues by accelerating or decelerating. |
| **Jump Rope** | **1-2 minutes** | **3** | Lights placed on the floor. Jump over lights as they illuminate sequentially. |

**Upper Body Exercises with FITLIGHT**

| **Exercise** | **Repetitions** | **Sets** | **FITLIGHT Integration** |
| --- | --- | --- | --- |
| **Push-Ups** | **10-15 reps** | **3** | Place lights on the floor near your hands. Touch lights with one hand while doing push-ups. |
| **Pull-Ups** | **5-10 reps** | **3** | Hang lights at different heights. Reach up to deactivate lights during pull-ups. |
| **Shoulder Press** | **10-12 reps** | **3** | Hold dumbbells with lights attached. Lift weights and deactivate lights above head. |
| **Dumbbell Curls** | **10-12 reps** | **3** | Attach lights to dumbbells. Perform curls and react to color changes on the lights. |

**Lower Body Exercises with FITLIGHT**

| **Exercise** | **Repetitions** | **Sets** | **FITLIGHT Integration** |
| --- | --- | --- | --- |
| **Squats** | **15-20 reps** | **3** | Place lights on the floor. Squat down to touch illuminated lights with your hands. |
| **Lunges** | **10-12 reps per leg** | **3** | Place lights on the ground. Step forward into a lung to deactivate lights. |
| **Leg Raises** | **12-15 reps** | **3** | Lights on the floor. Lift legs to point to toes at illuminated lights. |
| **Single Leg Deadlift** | **10-12 reps per leg** | **3** | Place lights on the floor. Extend leg backward while reaching for lights with hands. |

**Core and Stability Exercises with FITLIGHT**

| **Exercise** | **Repetitions / Duration** | **Sets** | **FITLIGHT Integration** |
| --- | --- | --- | --- |
| **Plank** | **30-60 seconds** | **3** | Place lights around hands/feet. Tap lights without breaking plank form. |
| **Sit-Ups** | **15-20 reps** | **3** | Place lights overhead. Perform sit-ups and reach to deactivate lights. |
| **Side Stretch** | **30 seconds per side** | **2** | Place lights on both sides. Stretch toward illuminated lights alternately. |
| **Back Extensions** | **12-15 reps** | **3** | Place lights ahead of body. Lift torso to deactivate lights during extensions. |

**Agility and Balance Exercises with FITLIGHT**

| **Exercise** | **Repetitions / Duration** | **Sets** | **FITLIGHT Integration** |
| --- | --- | --- | --- |
| **High Knees** | **30-60 seconds** | **3** | Place lights on the floor. Run in place and lift knees to hit lights. |
| **Box Jumps** | **8-10 reps** | **3** | Place lights on boxes/platforms. Jump onto platforms to deactivate lights. |
| **Balance Exercise** | **30-60 seconds per leg** | **2** | Stand on one leg. React to random light cues by shifting weight or stepping. |

**Cool Down Stretches with FITLIGHT**

| **Exercise** | **Duration** | **Sets** | **FITLIGHT Integration** |
| --- | --- | --- | --- |
| **Cool Down Stretch** | **5-10 minutes** | **1** | Use lights to guide stretches (e.g., reach for lights during hamstring or quad stretch). |
